# A Comparison of Transanal Drainage Tube and Diverting Ileostomy Following Low Anterior Resection for Rectal Cancer

**DOI:** 10.34172/mejdd.3518

**Published:** 2026-04-30

**Authors:** Masih Jahanbakhsh, Ahmed Mohammed Ali Hussein Alhurry, Sara Shojaei-Zarghani, Seyed Mohammad Kazem Tadayon, Mahboobeh Pourahmad, Mahshid Bahadori, Seyed Vahid Hosseini

**Affiliations:** ^1^Colorectal Research Center, Shiraz University of Medical Sciences, Shiraz, Iran; ^2^School of Medicine, Shiraz University of Medical Sciences, Shiraz, Iran; ^3^Department of General Surgery, Imam Hussein Medical City, Karbala, Iraq; ^4^Faghihi Hospital, Shiraz University of Medical Sciences, Shiraz, Iran

**Keywords:** Colorectal surgery, Rectal neoplasms, Low anterior resection syndrome, Quality of life

## Abstract

**Introduction:**

Diverting ileostomy (DS) and transanal drainage tube (TDT) are two common strategies to protect the anastomosis after low anterior resection (LAR). This study aimed to compare outcomes between patients receiving either a DS or a TDT following LAR.

**Methods:**

In this retrospective cohort study, we included eligible patients with rectal cancer who underwent LAR between March 2021 and February 2025 at our centers and had no history of neoadjuvant radiotherapy. Hospital length of stay, as a short-term outcome, and long-term outcomes-—including low anterior resection syndrome (LARS) score and quality of life (QoL)—were compared between the DS and TDT groups.

**Results:**

Among 74 eligible patients (52.7% men), 24 were in the TDT group and 50 in the DS group. Compared with the DS group, the TDT group had a significantly shorter hospital stay (*P*<0.001) and a better LARS score (*P*=0.004). Multivariable logistic regression analysis adjusted for age and sex revealed that DS was independently associated with increased odds of LARS (OR=4.92, 95% CI: 1.51–16.09). There were no significant differences between the groups regarding most QoL domains.

**Conclusion:**

For patients undergoing LAR for rectal cancer without neoadjuvant radiotherapy, TDT placement was associated with a shorter hospital stay and better long-term bowel function.

## Introduction

 Rectal cancer is the eighth most common cancer worldwide, with 729,833 new cases reported in 2022.^1^ Low anterior resection (LAR) is considered the gold standard surgical procedure for tumors in the mid-to-lower rectum, as it prevents the need for a permanent ileostomy.^2^ Surgeons frequently employ a diverting ileostomy (DS) as a fecal diversion strategy to protect the anastomosis and reduce postoperative complications.^3,4^ However, DS is associated with poor quality of life (QoL), stoma-related complications, and the need for stoma reversal surgery, which carries additional risks.^5-7^ Because of these challenges, the transanal drainage tube (TDT), a less invasive technique, has been proposed as an alternative to reduce intraluminal pressure. Several studies have compared short-term outcomes between DS and TDT. A previous meta-analysis found no significant differences between TDT and DS regarding anastomotic leak, surgical and infectious complications, or 30-day reoperation rates.^8^ Nevertheless, few studies have evaluated long-term outcomes, highlighting the need to shift the research focus from short-term complications toward functional outcomes following LAR.

 LAR surgery is frequently associated with persistent postoperative bowel dysfunction, including diarrhea, urgency, and fecal incontinence, collectively known as low anterior resection syndrome (LARS). LARS leads to substantial challenges for patients and significantly impairs long-term QOL among rectal cancer survivors. DS has been reported as an independent risk factor for LARS, potentially due to lower anastomotic levels and higher rates of radiotherapy in these patients,^9^ however, evidence remains conflicting.^10^ Therefore, the present study aimed to compare short-term (length of hospital stay) and long-term (LARS score and QoL) outcomes between patients who underwent placement of a TDT or DS following LAR without neoadjuvant radiotherapy.

## Materials and Methods

 In this retrospective cohort study, we enrolled all eligible patients who underwent LAR for rectal cancer at two major tertiary care centers in southern Iran Shahid Faghihi Hospital and Mother and Child Hospital in Shiraz between March 2021 and February 2025. Inclusion criteria included adult patients who underwent low rectal anastomosis with either a TDT or DS and had no history of preoperative radiotherapy. The selection of TDT or DS for anastomotic protection following LAR was determined by the operating surgeon based on patient-specific and surgical factors, including anastomotic height and intraoperative technical considerations. Informed consent was obtained from all participants prior to enrollment. The study protocol was approved by the Ethics Committee of Shiraz University of Medical Sciences (Approval No: IR.SUMS.MED.REC.1404.451) and conducted in accordance with the principles of the Declaration of Helsinki.

 Patient data, including age, sex, smoking status, comorbidities, surgical details, tumor characteristics, preoperative treatments, and postoperative complications, were extracted from the Shiraz Colorectal Cancer Surgery (SCORCS) registry. This ongoing database has collected information on patients with colorectal cancer undergoing surgery at our institutions since 2007.^11^ Supplemental data were obtained via telephone interviews. The QoL was assessed using the validated Persian version of the European Organization for Research and Treatment of Cancer Quality of Life Questionnaire-C30 (EORTC QLQ-C30).^12^ This questionnaire evaluates global health status, five functional scales (physical, role, emotional, cognitive, and social), multiple symptom scales (fatigue, nausea/vomiting, pain, dyspnea, appetite loss, insomnia, constipation, diarrhea), and the financial impact of the disease. Scoring was performed according to the official manual.^1^ LARS symptoms were evaluated using the corresponding 5-item questionnaire.^13^ Accordingly, patients were classified as having no LARS (0–20 points), minor LARS (21–29 points), or major LARS (30–42 points).

 The normality of quantitative variables was assessed with the Kolmogorov–Smirnov test. Normally distributed data are presented as mean ± standard deviation (SD) and were compared between the TDT and DS groups using the independent samples t-test. Non-normally distributed data are reported as medians with interquartile ranges (IQRs) and analyzed with the Mann–Whitney U test. Categorical variables are expressed as frequencies (percentages) and compared using the Chi-square test or Fisher’s exact test, as appropriate. Logistic regression was also conducted to assess the determinants of any LARS (minor or major), with results reported as odds ratios (ORs) and 95% confidence intervals (CIs). Variables with a *P*-value of less than 0.20 in the univariate analysis were included in the final multivariable model. All analyses were performed using SPSS software version 25, with a two-tailed *P* value < 0.05 considered statistically significant.

## Results

 During the study period, 31 patients with rectal cancer underwent LAR with a TDT insertion, while 343 patients underwent LAR with DS placement. From the DS group, 242 patients who had received neoadjuvant radiotherapy were excluded. Among all other eligible patients, 37 died, eight had missing data, 10 had a stoma at the time of questionnaire completion, and three were non-Iranian citizens. Consequently, 74 patients (52.7% men) were included in the analysis, consisting of 24 (32.4%) in the TDT group and 50 (67.6%) in the DS group. The baseline characteristics of the included patients are presented in Table 1.

**Table 1:** Patients’ characteristics

**Variable**	**Total**	**Diverting stoma**	**Rectal tube**	***P*** ** value**
n (%)	74	50 (67.6)	24 (32.4)	
Men, n (%)	39 (52.7)	26 (52.0)	13 (54.2)	0.861^‡^
Smoker/Ex-smoker, n (%)	18 (24.3)	9 (18.0)	9 (37.5)	0.067^‡^
Diabetes at surgery, n (%)	12 (16.2)	7 (14.0)	5 (20.8)	0.455^‡^
Hypertension at surgery, n (%)	20 (27.0)	11 (22.0)	9 (37.5)	0.160^‡^
Stage, n (%) Complete response I II III IV	10 (13.9) 22 (30.6) 14 (19.4) 25 (34.7) 1 (1.4)	9 (18.8) 13 (27.1) 7 (14.6) 18 (37.5) 1 (2.1)	1 (4.2) 9 (37.5) 7 (29.2) 7 (29.2) 0 (0.0)	0.216^¥^
Neoadjuvant chemotherapy, n (%)	19 (25.7)	18 (36.0)	1 (4.2)	0.004^¥^
Laparoscopy procedure, n (%)	65 (87.8)	42 (84.0)	23 (95.8)	0.256^¥^
Age at questionnaire completion (years), mean ± SD	60.41 ± 13.82	58.58 ± 13.37	64.39 ± 14.24	0.095^*^
Time since surgery (month), median (IQR)	31.00 (19.00-40.00)	30.50 (19.00-36.25)	33.00 (25.25-45.00)	0.044^†^
Size of tumor (cm), median (IQR)	3.50 (2.00-4.62)	3.00 (0.87-4.00)	4.00 (3.00-5.00)	0.074^†^
Distance to anal verge (cm), mean ± SD	9.93 ± 4.06	9.00 ± 3.48	13.83 ± 4.31	0.007^*^
Hospital stay (days), median (IQR)	7.50 (5.00-10.00)	9.00 (7.00-12.75)	5.50 (4.00-7.00)	< 0.001^†^

Abbreviations: IQR: interquartile range, SD: standard deviation. Parametric data are presented as mean ± SD, and non-normally distributed data as median (IQR). Categorical data are reported as frequencies (percentages). Between-group comparisons were conducted using independent-sample t-test*, Mann–Whitney U test^†^, Chi-square^‡^, or Fisher’s exact^¥^ tests, as appropriate. *P* values < 0.05 were considered significant.

 No significant differences were observed between the groups regarding sex, age, smoking status, comorbidities at surgery, type of surgery, tumor stage, or tumor size. However, a history of neoadjuvant chemotherapy was significantly more common in the DS group (36%) than in the TDT group (4.2%) (*P* = 0.004). Additionally, tumors in the DS group were located significantly closer to the anal verge (*P* = 0.007). Patients in the TDT group had a significantly shorter hospital stay compared to those in the DS group (*P* < 0.001).

 As presented in Table 2, significant differences in the LARS symptoms were observed between the groups, with the DS group exhibiting significantly worse LARS scores (*P* = 0.004). Multivariable logistic regression analysis, adjusted for age and sex, revealed that DS was independently associated with increased odds of LARS (OR = 4.92, 95% CI: 1.51–16.09, *P* = 0.008) (Table 3).

**Table 2:** Comparison of LARS between groups

**Variable, n (%)**	**Total**	**Diverting stoma (n=50)**	**Rectal tube (n=24)**	***P*** ** value**
No LARS	39 (52.7)	20 (40.0)	19 (79.2)	0.004
Minor LARS	19 (25.7)	15 (30.0)	4 (16.7)	
Major LARS	16 (21.6)	15 (30.0)	1 (4.2)	

Abbreviations: LARS: low anterior resection syndrome. Between-group comparison was conducted using Fisher’s exact test. *P* values < 0.05 were considered significant.

**Table 3:** Determinants of any LARS (minor/major)

**Variables **	**Univariate**	***P*** ** value**	**Multivariable**	***P*** ** value**
Age at surgery	0.96 (0.93-0.99)	0.039	0.97 (0.93-1.00)	0.085
Sex (Female *vs*. Male)	2.13 (0.84-5.40)	0.110	2.55 (0.90-7.21)	0.078
Group (stoma *vs.* rectal tube)	5.70 (1.83-17.75)	0.003	4.92 (1.51-16.09)	0.008
Distance to anal verge (cm)	0.90 (0.75-1.09)	0.291		
Neoadjuvant chemotherapy (Yes *vs*. No)	1.00 (0.35-2.85)	0.994		

Data are reported as odds ratios (ORs) and 95% confidence intervals (CIs). Variables with a *P* value of less than 0.20 in the univariate analysis were included in the final multivariable model. Abbreviations: LARS: low anterior resection syndrome *P* values < 0.05 were considered significant.

 Regarding QoL domains, no significant differences were observed between the groups except for insomnia, which was more frequently reported in the TDT group (Table 4, *P* = 0.027).

**Table 4:** Comparison of quality of life according to the EORTC QLQ-C30 questionnaire between groups

**Variable**	**Diverting stoma (n=50)**	**Rectal tube (n=24)**	***P*** ** value **
Global health status	75.00 (64.58-83.33)	79.17 (58.33-97.92)	0.407
Physical functioning	86.67 (80.00-93.33)	86.67 (68.33-98.33)	0.639
Role functioning	100.00 (95.83-100.00)	100.00 (83.33-100.00)	0.775
Emotional functioning	83.33 (66.67-100.00)	87.50 (52.08-100.00)	0.850
Cognitive functioning	100.00 (83.33-100.00)	91.67 (83.33-100.00)	0.297
Social functioning	100.00 (83.33-100.00)	100.00 (87.50-100.00)	0.776
Fatigue	11.11 (0.00-22.22)	22.22 (0.00-52.78)	0.131
Nausea and vomiting	0.00 (0.00-00.00)	0.00 (0.00-0.00)	0.421
Pain	16.67 (0.00-33.33)	16.67 (0.00-33.3)	0.818
Dyspnea	0.00 (0.00-0.00)	0.00 (0.00-0.00)	0.734
Insomnia	0.00 (0.00-33.3)	33.33 (0.00-66.67)	0.027
Appetite loss	0.00 (0.00-0.00)	0.00 (0.00-50.00)	0.583
Constipation	0.00 (0.00-33.33)	33.33 (0.00-33.33)	0.170
Diarrhea	0.00 (0.00-0.00)	0.00 (0.00-0.00)	0.453
Financial difficulties	33.33 (0.00-66.67)	0.00 (0.00-58.33)	0.187

Abbreviations: EORTC QLQ-C30: European Organization for the Research and Treatment of Cancer Quality of Life Questionnaire, IQR: interquartile range. Data are reported as median (IQR). Between-group differences were determined using the Mann–Whitney U test. *P* values < 0.05 were considered significant.

## Discussion

 This study compared short- and long-term outcomes between TDT and DS following LAR for rectal cancer in patients without neoadjuvant radiotherapy. Our findings demonstrate that TDT provides comparable QoL while being associated with a significantly better LARS score and shorter hospital stay.

 The worse LARS scores observed in the DS group are consistent with the existing literature linking fecal diversion to long-term bowel dysfunction, potentially mediated by diversion colitis, alterations in gut microbiota, and delayed neorectal adaptation^14-17^ Furthermore, a lower anastomotic level has been reported as a significant risk factor for LARS.^18^ As expected, patients in the DS group had a significantly lower tumor distance to the anal verge compared with those in the TDT group, which may play a key role in the increased risk of LARS. However, we found no association between neoadjuvant chemotherapy or distance to the anal verge and any LARS in the univariate analysis. The current literature regarding the effects of TDT on postoperative bowel function remains limited and warrants further investigation in future studies.

 Despite worse LARS scores in the DS group, most QoL domains were statistically similar between the two groups. Although a recent meta-analysis reported significant negative effects of LARS on QoL,^19^ several other studies have reported findings similar to ours.^20,21^ The lack of a significant association between LARS severity and QoL in the present study may reflect patients’ psychological adaptation over time, as well as the influence of multiple factors, such as psychological and social dimensions, in addition to bowel dysfunction on QoL. Notably, insomnia was more frequent in the TDT group, suggesting that sleep disturbances may represent an underrecognized long-term outcome in this population. However, this finding should be interpreted with caution, as it may represent a chance occurrence due to multiple comparisons. Future prospective studies are warranted to longitudinally assess psychological characteristics and sleep-related outcomes over time.

 Several limitations of this study should be acknowledged. First, the retrospective design introduces the potential for selection bias. Significant baseline differences were observed between the groups, particularly regarding tumor distance from the anal verge and neoadjuvant chemotherapy. Second, the limited sample size reduced statistical power and precluded extensive multivariable adjustment. Third, our findings may have limited generalizability to other populations, as the study was conducted at two centers in southern Iran and included only patients who had not received neoadjuvant radiotherapy. Finally, long-term oncological outcomes and postoperative complications were not assessed. Future multicenter prospective randomized controlled trials with larger sample sizes and extended follow-up periods are warranted to confirm our findings.

## Conclusion

 In patients undergoing LAR for rectal cancer without neoadjuvant radiotherapy, TDT offered a shorter hospital stay and improved long-term bowel function. Further studies should be conducted to better elucidate the potential of TDT as an effective alternative to routine DS in carefully selected patients.
